# Health promoting lifestyles and influencing factors among empty nesters and non-empty nesters in Taiyuan, China: a cross-sectional study

**DOI:** 10.1186/s12955-018-0936-5

**Published:** 2018-05-25

**Authors:** Chichen Zhang, Ruifang Zhu, Jiao Lu, Yaqing Xue, Lihong Hou, Mimi Li, Xiao Zheng, Tingzhong Yang, Jianzhong Zheng

**Affiliations:** 1 School of Management, Shanxi Medical University, Taiyuan, Shanxi China; 2 School of Nursing, Shanxi Medical University, Taiyuan, Shanxi China; 3 Center for Tobacco Control Research, Zhejiang University School of Medicine, Zhejiang, Hangzhou China

**Keywords:** Health behavior, Influencing factors, Elderly, Cross-sectional survey

## Abstract

**Background:**

In China, the problems of population aging and empty nesting have become important issues which will affect the social stability and economic development. The aim of this study was to explore the health promoting lifestyles and influencing factors among empty nesters and compare with non-empty nesters to find out their differences, so as to provide a scientific evidence for people to formulate health management strategies for elderly.

**Methods:**

A cross-sectional survey which used a stratified random cluster sampling method, was conducted among 500 elders in six districts of Taiyuan, China, there were 288 empty nesters and 212 non-empty nesters. The general information and health- promoting lifestyles were investigated by using the self-made General Information Questionnaire and Health Promoting Lifestyle Scale(HPLP). Two-sample *t*-test and Chi-square test were used to compare the sociodemographic factors, HPLP scores of empty nesters to non-empty nesters; Multiple stepwise linear regression was performed to estimate influencing factors related to the HPLP of empty nesters and non-empty nesters.

**Results:**

The current findings showed that there were differences between the empty nesters and non-empty nesters in gender, resident, marital status, education and income, self-care ability, source of income, relationship with spouse and social activities (*P* < 0.05). Empty nesters were mostly male, married, had a higher education level, self-care ability and income and lived in urban compared with non-empty nesters. The health promoting lifestyles of the elderly in this survey were in the medium level, the highest score for all dimensions in both groups was in nutrition, whereas health responsibility was executed worst. The HPLP and six subscales scores of the empty nesters were higher than non-empty nesters, there were significant differences in total score of HPLP, self-realization and health responsibility (*P* < 0.01). Multiple regression analysis showed that the main predictive factors for the empty nesters were education, self-care ability and resident, whereas the main predictive factors for the non-empty nesters were parents-child relationship, source of income and age; social activity was the common factor for two group.

**Conclusion:**

The health promoting lifestyles of the empty nesters was better than that of the non-empty nesters. Health responsibility, interpersonal relations and stress management were key dimensions to be improved. Except social activity, education, self-care ability and resident were the unique influencing factors of health-promoting lifestyles for empty nesters, while the parents-child relationship, income and age were unique factors for non-empty nesters. The main target of Intervention strategy for elderly health promoting lifestyles should be the enhance of health responsibility, interpersonal relations and stress management by improving social activities, parent-child relationship, education and income of elderly.

## Background

The aging is a universal problem for all the world, because of the changing of population structure in China, the population aging playing a prominent role, one of the most iconic is the increasing of empty nesters. A survey conducted by The Sixth National Census in 2010 showed that 13.3% of the total population was aged 60 and above in China. It indicates that the aging process gradually speeds up, meanwhile, the number of empty nest families is increasing [1]. In 2014, the survey of National Committee on Ageing determined that empty nesters accounted for 51.1% of the elderly in China [2]. It is estimated that the proportion of empty-nesters households will reach 90% by 2030 [3], when all of the elderly families will have “an empty nest”. The empty nest is becoming the main family pattern in China. In China, an empty nest family is one in which an elderly person lives alone or with his or her spouse. The children have left home like birds flying away from the nest, and the elderly parents are left alone and do not receive the care of their children [4]. The elderly are vulnerable to various diseases and health problems, therefore, it is essential to find an effective way to improve the health of elderly.

Professor Pender, an American nursing scientist, proposed the concept of a “health promoting lifestyles” in 1987 [5]. According to that concept, self-initiated and multi-level behaviors and perceptions are undertaken by an individual to maintain or increase his health level and to achieve self-actualization and self-satisfaction. The concept includes six aspects: health responsibility, self-actualization, interpersonal support, nutrition, sports and pressure management. Health promoting lifestyles can indeed free one from many diseases and help one enjoy a better life. Previous studies have shown that individuals following a health promoting lifestyles were healthier and suffered less from the pains of diseases [6, 7]. There were correlations between health-promoting lifestyles and the psychological, social, and physical health of Chinese elderly [8, 9]. Yan [8] found that the health promoting lifestyles of urban elderly were closely related to depression. Yin [7] found that elderly with a strong awareness of health-promoting lifestyles were healthier than those without. Based on the above studies, the improvement of health promoting lifestyles could enhance the health of elderly.

Excising researches pay more attention to the health promoting lifestyles of elderly, but few on empty nesters. Meanwhile, there were few reports comparing the health promoting lifestyles of non-empty nesters and empty nesters. The correlations and differences between the groups are still unclear. The study assumed that there are differences in health promoting lifestyles for elderly under different family structure. Non-empty nesters who live with their children, can get more care, so we assumed their health promoting lifestyles were better than that of empty nesters. The current research attempts to dig out the health promoting lifestyles of empty nesters and non-empty nesters, and compare the differences of them, even the influencing factors.

Some scholars have also studied influencing factors of the elderly people’s health promoting lifestyles. Research carried out by Bu et al. [10]. showed that the elderly peoples’ overall level of health promoting lifestyles in urban communities was high, with nutrition level being the highest. They also noted that marital status and household income were two major influencing factors of the elderly peoples’ lifestyles. Sun [11] conducted a survey of nursing institutions for the elderly in Xi’an, and their results suggested that health promoting lifestyles were at a low level. Likewise, they found that marital status, economic income and degree of education were major factors impeding the local popularization of health promoting lifestyles. Therefore, this study assumed that marital status, income, and education were the common influencing factors for health promoting lifestyles of empty nesters and non-empty nesters. Meanwhile, source of income, social activities, parents-child relationship and relationship with spouse were included in this study. However, the specific differences between the two groups need to be further analyzed.

## Methods

### Study design and participants

A questionnaire-based cross-sectional study was conducted in Taiyuan, which is the capital in Shanxi Province and have six districts (Ying ze, Xiao dian, Xing hualing, Wan bailin, Jin yuan and Jian caoping). The report by Civil affairs bureau of Taiyuan in 2014 showed that there were 638,000 people over 60 years old in Taiyuan, accounting for 14.8% of the total population, in which there were 350,000 empty nesters, accounting for 54.8%, and 2,0000 were disabled or partially disabled [12].

The sample size was estimated according to the following formula $\mathrm{N}=\frac{u_{\upalpha /2}^2\pi \left(1-\pi \right)}{\delta^2}$ (π: overall rate) [13]. Previous research showed that the rate of medium health promoting lifestyles of the elderly in China ranged a large gap from 40 to 80% [8, 14, 15], so in this study, π = 50%, *u*_α/2_ = 1.96, δ=0.1π = 0.05. The sample size was determined to be 385. A stratified random cluster sampling method was used. First, according to the order of communities on the government’s website, each community in every district was numbered. Next, two communities were randomly selected in each district using a random-number table, which can draw random sampling scientifically. Third, a residential district was randomly chosen from each selected community using the same method. Forth, residential buildings were randomly chosen from each selected residential district. All the elderly living in the selected residential building were enrolled in this study. The types of residential buildings in Taiyuan were different, which accommodated 12 to 105 families. In order to ensure adequate samples, if a building did not have enough elderly living in it, another building in the residential district was randomly selected to supplement it. A total of 21 buildings were surveyed, included 7–47 elderly in each building, average 25 elderly/building. Elders who were aged 60 years and above, could communicate in Chinese and had no cognitive disorders were eligible for the study. Elders were excluded from the study if they had cognitive disorders or serious diseases, such as deafness, psychiatric disorders or Alzheimer’s disease [16].

All participants were informed of the study procedure upon their recruitment. After obtaining written consent, participants were interviewed by trained study interviewers. For the participants who were in poor health or had limited understanding and expression, written consent was obtained from their children or legal guardians. All study procedures were approved by the Ethics Committee of Shanxi Medical University.

This study was performed from June 2015 to January 2016. Of the 530 individuals contacted, 500 completed the questionnaires. The response rate was 94.3%.

### Instruments

There were 2 study self-administered questionnaires or scales involved in the study: (1) a basic information questionnaire, which was asked for age, gender, residence, current marital status, family structure, education, economic and chronic disease, social activities, self-care ability, source of income, relationship with children or spouse. The elderly answered the questionnaire according to their own socioeconomic status and lifestyles, in which we found the factors influencing the health promoting lifestyles of empty nesters and non-empty nesters. (2) the health-promoting lifestyle profile (HPLP-C) [5, 17], revised by Huang from Taiwan Zhongshan University, which consisted of 6 dimensions and 42 items, including self-realization, health responsibility, physical activity, nutrition, interpersonal relations, and stress management. Each item assessed symptom severity on a 4-point Likert scale. Health promoting lifestyles was defined by the total score, with higher score indicating a better health promoting lifestyles. Based on single split for scale, the health promoting lifestyles can be divided into 3 levels: high (3–4 points), medium (2–3 points), and low (1–2 points). HPLP Scale has been proven to have a good reliability, Cronbach’α of the total scale and 6 dimensions ranging from 0.74 to 0.93 [18]. In the studied population, the internal consistency of the scale was high, overall Cronbach’s α = 0.91, with all 6 dimensions ≥ 0.41, and correlation coefficients ranging from 0.36 to 0.87.

### Statistical

EpiData was used to enter and check the validity of data, and SPSS20.0 was employed to analyze data. Measurement data were presented as ($\overline{x}\pm s$). The Chi-square test and Two-sample *t*-test were used to compare the sociodemographic and health promoting lifestyles differences of empty nesters and non-empty nesters. Using multiple stepwise linear regression to explore influence factors of health promoting lifestyles. All tests were two-sided, with α = 0.05.

## Results

There were 288 (57.6%) empty nesters and 212 (42.4%) non-empty nesters. The chi-squared test showed that there were significant differences between the empty-nesters and non-empty nesters in gender, resident, marital status, education and income, self-care ability, source of income, relationship with spouse and social activities (*P* < 0.05). This result meant that the empty nesters were mostly male, married, had a higher education level, self-care ability and income and lived in urban areas compared with non-empty nesters (Table 1).

**Table 1:** Demographic characteristics of the empty nesters and the non-empty nesters

Characteristics	Empty-nesters		Non-empty nesters		*χ2*	*P*
	N	%	N	%		
Gender					8.65	0.003
Male	170	59.03%	97	45.75%		
Female	118	40.97%	115	54.25%		
Age					3.17	0.205
Low (60–69 years)	155	53.82%	131	61.79%		
Middle (70–79 years)	94	32.64%	57	26.89%		
High (80 years or above)	39	13.54%	24	11.32%		
Resident					31.36	< 0.001
Urban	231	80.21%	121	57.08%		
Rural	57	19.79%	91	42.92%		
Marital status					10.24	0.017
Married	224	77.78%	178	83.96%		
Discoverture	11	3.82%	0	0.00%		
Divorced	7	2.43%	2	0.94%		
Widowed	46	15.97%	32	15.10%		
Education					22.89	< 0.001
No education	29	10.07%	26	12.27%		
Primary school	58	20.14%	49	23.11%		
Secondary school	75	26.04%	84	39.62%		
High school	66	22.92%	35	16.51%		
Junior college	31	10.76%	11	5.19%		
Post-secondary school and above	29	10.07%	7	3.30%		
Income					11.82	0.008
No	45	15.63%	56	26.42%		
1000 and below	50	17.36%	35	16.51%		
1000~	104	36.11%	77	36.32%		
3000~	89	30.90%	44	20.75%		
Chronic disease					0.31	0.579
Yes	139	48.26%	97	45.75%		
No	149	51.74%	115	54.25%		
Self-care ability					7.37	0.025
Complete	251	87.15%	200	94.34%		
Portion	28	9.72%	10	4.72%		
Unable	9	3.13%	2	0.94%		
Source of income						
Pension	189	65.62%	97	45.75%	21.40	0.001
Income by work	40	13.89%	42	19.81%		
Supply by children	32	11.11%	34	16.04%		
Supply by spouse	8	2.78%	12	5.66%		
Social relief	14	4.86%	17	8.02%		
Others	5	1.74%	10	4.72%		
Parents-child relationship					5.35	0.253
Beautifully	4	1.39%	0	0.00%		
Good	103	35.76%	81	38.21%		
General	166	57.64%	122	57.54%		
Little bad	12	4.17%	9	4.25%		
Bad	3	1.04%	0	0.00%		
Relationship with spouse					11.84	0.019
Beautifully	11	3.82%	3	1.42%		
Good	88	30.55%	85	40.09%		
General	181	62.85%	116	54.72%		
Little bad	3	1.04%	7	3.30%		
Bad	5	1.74%	1	0.47%		
Social activities					9.76	0.021
More	19	6.60%	6	2.83%		
Many	84	29.17%	47	22.17%		
Little	136	47.22%	106	50.00%		
No	49	17.01%	53	25.00%		

### HPLP subscales scores among empty nesters and non-empty nesters

Health-promoting lifestyle was divided into three levels according to each item: high (3–4 points), medium (2–3 points) and low (1–2 points). For most empty nesters (72.6%) and non-empty nesters (70.75%), the health-promoting lifestyle were in the medium level; the highest score for all dimensions in both groups was in nutrition, followed by self-realization, physical activity, stress management and interpersonal relations. The lowest score was in health responsibility. For both empty nesters and non-empty nesters, nutritional behavior was executed best, whereas health responsibility was executed worst. Meanwhile, the total and average scores on the six subscales were higher for empty nesters than they were for non-empty nesters. The two-sample *t-*test showed that there were significant differences between empty nesters and non-empty nesters in total score of HPLP, self-realization and health responsibility (*P* < 0.01). This result suggested that the overall health promoting lifestyles of empty nesters was superior to that of non-empty nesters (Table 2).

**Table 2:** HPLP subscale scores among empty nesters and non-empty nesters ($\overline{x}\pm s$)

Characteristics	Empty nesters	Rank	Non-empty nesters	Rank	*t*	*P*	*95%CI*
HPLP	104.20 ± 19.23		98.73 ± 18.53				
Average	2.48 ± 0.46		2.35 ± 0.44		3.19	0.002	(0.050~0.210)
Self-realization	2.68 ± 0.61	2	2.54 ± 0.58	2	2.65	0.008	(0.037~0.248)
Health responsibility	1.95 ± 0.57	6	1.73 ± 0.55	6	4.41	< 0.001	(0.123~0.322)
Physical activity	2.57 ± 0.92	3	2.43 ± 0.95	3	1.64	0.102	(−0.028~0.303)
Nutrition	3.04 ± 0.47	1	3.03 ± 0.43	1	0.17	0.864	(−0.073~0.872)
Interpersonal relations	2.30 ± 0.68	5	2.20 ± 0.79	5	1.55	0.123	(−0.025~0.233)
Stress management	2.44 ± 0.58	4	2.36 ± 0.56	4	1.60	0.110	(−0.019~0.186)

### Analysis in influencing factors of health promoting lifestyles among empty nesters and non-empty nesters

To compare the influencing factors of HPLP in the empty nesters and non-empty nesters, sociodemographic variables were defined as independent variable X and dependent variable Y in HPLP to perform stepwise linear regression analysis. The inclusion criterion was 0.05, and the exclusion criterion was 0.10.

In the empty nesters, the results showed that degree of education, involvement in social activities, self-care ability and resident were predictive factors. All factors presented a positive correlation with the health promoting lifestyles of empty nesters. Thus, higher education, more social activities, and better self-care ability were related to higher HPLP. Meanwhile, empty nesters living in urban areas had a better HPLP than those in rural areas (Table 3).

**Table 3:** Multiple stepwise linear regression on the relationship between sociodemographic variables and HPLP among empty nesters

Independent variables	Estimate (B)	Standardized estimate (*β*)	*P*	95%CI
Social activities	−5.47	0.18	0.001	(−8.733~ −2.211)
Education	2.73	0.20	< 0.001	(1.206~4.245)
Self-care ability	−6.11	0.14	0.009	(−10.676~ −1.533)
Resident	−6.79	0.14	0.013	(−12.166~ −1.416)

Model *R*^2^ = 0.228

As shown in (Table 4), the results indicated that the parents-child relationship, source of income, social activities and age were related to non-empty nesters. Specifically, according to the parents-child relationship, the score increased by 0.27 with an increase in level. Stable income means high scores, for example, an individual with personal income or their income from their children had a higher score than people who receiving social relief. The higher involvement in social activities was, the higher the score was. For every 10 years that a non-empty nester aged, the score reduced by 0.17.

**Table 4:** Multiple stepwise linear regression on the relationship between sociodemographic variables and HPLP among non-empty nesters

Independent variables	Estimate (B)	Standardized estimate (β)	*P*	95%CI
Source of income	−2.77	0.23	< 0.001	(−4.307~ −1.233)
Parents-child relationship	−9.01	0.27	< 0.001	(−12.886~ −5.133)
Social activities	−4.99	0.21	0.001	(−7.833~ −2.139)
Age	4.59	0.17	0.005	(1.437~ 7.738)

Model *R*^2^ = 0.296

## Discussion

In this study, the health-promoting lifestyles of empty nesters were better than those of non-empty nesters.

The current findings showed that there were differences between empty nesters and non-empty nesters in terms of gender, resident, marital status, education and income. Gender research suggests that males play three roles in the world: professional, social and house roles [19, 20]. After retirement, males’ professional and social roles weaken. The traditional patriarchal concept in China makes males reluctant to live with their offspring, so there are no opportunities for them to reshape their house roles. In contrast, females consider the house role to be their major role, they think that it is their responsibility to take care of their offspring. Thus, there were more male empty nesters than there were females. Moreover, the proportion of the empty nesters living in urban areas was higher than the proportion of those living in rural areas, and the former’s education level was higher. Perhaps because people living in urban areas have more access to education, for them, the traditional concept of “not traveling far away when your parents are still alive” has be changed. Moreover, compared with their rural counterparts, people in urban areas have more employment opportunities. Fixed incomes, such as pensions, allow urbanites to live an independent life without relying on their offspring.

Our study also found that among the six health promoting lifestyles, the nutrition of empty nesters and non-empty nesters were the best, whereas the health responsibilities were the worst. These results were similar to others from diverse population groups, such as Thailand physical therapy students and Chinese community-dwelling adults [21]. As one grows old, the physical functions deteriorate, thus increasing the risk of disease. Because of health and disease treatment requirements, the elderly paid more attention to nutrition. However, their self-health management awareness were weak. A strong awareness of health responsibility has not yet been formed among them. Compared with non-empty nesters, the health promoting lifestyles of empty nesters were better. They have a strong awareness of self-actualization and health responsibility. Most empty nesters have probably retired and lived with their spouses or alone. We assumed that non-empty nesters who live with their children can get more care from their children, then their health promoting lifestyles were better. However, they were more likely be the caregiver for their children and offspring conversely. Without work pressure or the burden of taking care of youngsters, empty nesters have more time and energy to communicate with others and learn about maintaining their health.

The predictive factors must be found to improve the health promoting lifestyles of elderly. In our study, except social activities, the factors or indicators of a health-promoting lifestyles were different among empty nesters and non-empty nesters. As “social beings,” empty nesters and non-empty nesters both need to participate in social activities. Some literatures showed that social participation was related to better health-related quality of life [22], functional skills [23], and even survival [24]. Inadequate health literacy was a common problem among older adults and was associated with poor health outcomes [25]. Meanwhile, The elderly can communicate with others in activities, which is a good way for them to improve their behaviors through mutual exchanges and learning to improve their health. Local governments and communities should help elders participate in health-promoting activities, strengthen the publicity of health education, advocate healthy behavior, integrate health maintenance knowledge into their daily lives, and reduce the occurrence rate of dangerous factors.

For empty nesters, education and self-care ability were major factors influencing their health promoting lifestyles. Self-care ability is a basic activity of daily life, and because empty nesters live alone, they need to have more self-reliant ability. Previous studies reported the rate of morbidity in homebound elderly Chinese community was found to be 15.49% and it gradually increased with age, and also with a lower education or poorer activities of daily living (ADL) [26]. Higher education was often accompanied by high incomes, which tend to better health. Additionally, the elderly with a lower education level were more likely to be misled by negative information and to form unhealthy living habits. In urban areas, economic and cultural development is faster, infrastructures and services are more complete, and knowledge is spread more efficiently. Thus, empty nesters living in urban areas have more chances to receive education and quality services, and they are more likely to have a retirement pension; thus, they have the economic ability to live independently and enjoy their own life.

Compared with empty nesters, non-empty nesters rely on external factors for their health-promoting lifestyles, including income, income sources and relationships with their children. Research has revealed that family relationships have a close contact with elderly peoples’ health status, quality of life and death rate [27]. Family relationships include financial and emotional support. Non-empty nesters live with their offspring, have a closer connection with them and were influenced more by them. Having family around means that they have more opportunities for emotional communication with their children and can get help from them. Therefore, a harmonious family relationship can contribute to the formation of a health-promoting lifestyles. Meanwhile, a higher income means more economic independence and better ability to pay daily and medical expenses. However, most non-empty nesters have a low income. They rely on their offspring for economic support and do not have adequate income to enjoy their lives. This economic burden prevents them from improving their lifestyles and health. Hence, it is necessary to advocate vigorous social support and a harmonious family environment and to build a complete social security system for the elderly, so they can live in a favorable family environment. Without these worries, they can devote more time and energy to improving their health promoting lifestyles.

The results contradicted the assumption, the health promoting lifestyles of empty nesters were better than that of non-empty nesters. Social activities was the common influencing factors of health promoting lifestyles for the two groups, source of income, parents-child relationship and age were unique factors for non-empty nesters, and education, self-care ability and resident for empty nesters.

This study had some limitations. First, the study included the elderly participants from Taiyuan in Shanxi province, thus limiting representation. Second, the low literacy level and advanced age of the study population might have caused participants to misunderstand the questions or give inaccurate responses.

## Conclusion

In our study, the health promoting lifestyles of empty nesters were superior to non-empty nesters, health responsibility, interpersonal relations and stress management were key dimensions to be improved and the influencing factors on the both of them were different. These differences should be considered to direct health education activities. Parents-child relationship was the main factor of non-empty nesters for their health promoting lifestyles, so policymakers should pay more attention to non-empty nesters’ families and their intimacy with their offspring, which can help mediate their family conflicts. Education and social activities were main factors of empty nesters for their health promoting lifestyles, more social support should be given to empty nesters, and community cultural facilities and activities should be enriched. In this way, the elderly can fundamentally change their concepts of health, implement health-promoting lifestyles.

This study provided a new perspective to examine the health promoting lifestyles of the elderly, which provided a scientific basis for government and community workers to make promoting strategies for the elderly of their health promoting lifestyles. In the future study, it is essential to verify the results by expanding the sample size, comparing the differences between empty nesters and non-empty nesters in their health promoting lifestyles and influencing factors in different economic levels, and combined with interview or other qualitative research based on quantitative research to get a more scientific result.

## Abbreviations

GDP: Gross Domestic Product
HPLP: Health-promoting lifestyle

## References

[1] Wang GJ, Hu M, Xiao SY, Zhou L (2017). Loneliness and depression among rural empty-nest elderly adults in Liuyang, China: a cross-sectional study. BMJ Open.

[2] Ye C, Guo X, Liang G, Zhao L, Yang H, Yu S (2016). Comprehensive comparison between empty Nest and non-empty Nest elderly: a cross-sectional study among rural populations in Northeast China. Int J Environ Res & Public Health.

[3] Chen F, Xu XL, Yang Z, Tan HW, Zhang L (2015). The willingness-to-pay for general practitioners in contractual service and influencing factors among empty nesters in Chongqing. China Int J Environ Res Public Health.

[4] Jensen LW, Decker L, Andersen MM (2009). Depression and health-promoting lifestyles of persons with mental illnesses. Issues Mental Health Nurs.

[5] Walker SN, Sechrist KR, Pender NJ (1987). The health-promoting lifestyle profile: development and psychometric characteristics. Nurs Res.

[6] Liu Y, Liu L, Li YF, Chen YL (2015). Relationship between health literacy, health-related behaviors and health status: a survey of elderly Chinese. Int J Environ Res Public Health..

[7] Yin Z, Geng G, Lan X, Zhang L, Wang S, Zang Y (2013). Status and determinants of health behavior knowledge among the elderly in China: a community-based cross-sectional study. BMC Public Health.

[8] Hua Y, Wang B, Wallen GR, Shao P, Ni C, Hua Q (2015). Health-promoting lifestyles and depression in urban elderly Chinese. PLoS One.

[9] Umberson D, Crosnoe R, Reczek C (2010). Social relationships and health behavior across life course. Annu Rev Sociol.

[10] Bu XM, Su LR, Cao LJ (2005). Health-promoting lifestyle and the influencing factors in elderly people of urban community. Chin J Clin Rehabil.

[11] Sun L, Hua QZ, Zhou GS, Sun MJ, Zhang P (2013). Survey on health-promoting lifestyles among aged people living in nursing homes of Xi’an. J Nurs Sci.

[12] Li P. In: There were 638 thousand people over 60 years old in Taiyuan. Office of the China committee on aging. http://www.cncaprc.gov.cn/contents/37/21109.html. Accessed 24 Sept 2014.

[13] Sun ZQ. Medical Statistics (Third Edition). In: Sun ZQ, Xu YY, editors. People's Medical Publishing House(PMPH). China: Beijing; 2002. p.110–178.

[14] Wang SJ, Qiao D, Hao Y, Zhang Y, Liu MC, Li PF (2016). Health-promoting lifestyle among the elderly living in nursing homes. Baoding Modern Preventive Med.

[15] Zhang P, Zhao X, Zhao YN, Jing LW, Liu HJ, Bai H (2014). Health-promoting lifestyle and related factors among rural residents in Hebei province. Chin J Public Health.

[16] Guo YQ, Zhang CC, Huang H, Zheng X, Pan XJ, Zheng JZ (2016). Mental health and related influencing factors among the empty-nest elderly and the non-empty-nest elderly in Taiyuan, China: a cross-sectional study. Public Health.

[17] Huang YH, Chiou CJ (1996). Assessment of the health - promoting lifestyle profile on reliability and validity. Kaohsiung J Med Sci.

[18] Wang XM, Xie H (2013). Correlation of health promoting lifestyle and college adaptability among medical undergraduates. Chin J Sch Health.

[19] Chen H (2006). On the causes of the conflict of the double roles of Chinese city career women and some solutions. J Inner Mongolia Univ Technol (Social science edition).

[20] Wu ZQ, Cui GY, Zhang XJ, Sun YH (2009). Research on family function of elderly and its influential factors in Anhui province. Chin J Public Health.

[21] Nualnetr N, Thanawat T (2012). Health-promoting behaviors of physical therapy students. J Phys Ther Sci.

[22] Szanton SL, Roberts L, Leff B, Walker JL (2016). Home but still engaged: participation in social activities among the homebound. Qual Life Res.

[23] James BD, Boyle PA, Buchman AS, Bennett DA (2011). Relation of late-life social activity with incident disability among community-dwelling older adults. J Gerontol Ser A Biol Med Sci.

[24] Hsu HC (2007). Does social participation by the elderly reduce mortality and cognitive impairment?. Aging Ment Health.

[25] Geboers B, de Winter AF, Luten KA, Jansen CM, Reijneveld SA (2014). The association of health literacy with physical activity and nutritional behavior in older adults, and its social cognitive mediators. J Health Commun.

[26] Jing LW, Wang FL, Zhang XL, Yao T, Xing FM (2017). Occurrence of and factors influencing elderly homebound in Chinese urban community: a cross-sectional study. Medicine.

[27] Zhang Z (2002). The impact of intergenerational support on mortality of oldest old in China. Popul Res.

